# Brassinosteroids and gibberellic acid actively regulate the zinc detoxification mechanism of *Medicago sativa* L. seedlings

**DOI:** 10.1186/s12870-023-04091-4

**Published:** 2023-02-04

**Authors:** Ying Ren, Xue Li, Jingwei Liang, Sijia Wang, Zhihao Wang, Hui Chen, Ming Tang

**Affiliations:** State Key Laboratory of Conservation and Utilization of Subtropical Agro-Bioresources, Guangdong Laboratory for Lingnan Modern Agriculture, Guangdong Key Laboratory for Innovative Development and Utilization of Forest Plant Germplasm, College of Forestry and Landscape Architecture, South China Agricultural University, Guangzhou, 510642 China

**Keywords:** Zn toxicity, Gibberellic acid, Brassinosteroids, Heavy metal

## Abstract

**Background:**

Zinc is one of the essential trace elements in plants. There are few studies on the phytohormone to rescue the toxicity of excessive zinc to plants. The aim of this research was to evaluate the alleviating effects of brassinosteroids (BR) and gibberellic acid (GA) on the toxicity of *Medicago sativa* L. (*M. sativa*) induced by excessive zinc.

**Results:**

After zinc, BR and GA were applied to *M. sativa* seedlings for 7 weeks, their physiological and biochemical properties and gene expression patterns were evaluated. BR and GA significantly weakened the inhibition effect of zinc stress on growth and biomass of *M. sativa*. Under zinc stress, the zinc accumulation in *M. sativa* roots was over 5 times that in shoots. Application of BR and GA reduced zinc accumulation in roots. The content of lipid peroxides in *M. sativa* decreased and the activity of antioxidant enzymes increased under BR and GA treatments. In addition, BR and GA treatment down-regulated the transcription level of MsZIP1/3/5, the transporters of zinc uptake in root cells. And BR and GA up-regulated the expressions of zinc efflux, chelation, vacuolar storage and long-distance transport related genes: *MsZIP7*, *MsHMA1*, *MsZIF1*, *MsMTP1*, *MsYSL1* and *MsNAS1*.

**Conclusions:**

Our findings further showed that BR and GA application to *M. sativa* under zinc stress can reduce zinc accumulation, promote the response of the antioxidant defense system, and actively regulate the mechanism of heavy metal detoxification. Notably, 100 nM BR performed slightly better than 100 nM GA in all aspects of the detoxification of *M. sativa* by excessive zinc.

**Supplementary Information:**

The online version contains supplementary material available at 10.1186/s12870-023-04091-4.

## Introduction

Zinc (Zn) is an essential trace element in plants and a typically transition metal for the synthesis and action of various enzymes, auxin, and chlorophyll [1, 2]. However, excessive Zn can poison plants by inhibiting seed germination and root elongation, reducing photosynthesis and respiration, and damaging plant cell membranes, proteins and genetic material [1, 3–6]. And plant-enriched Zn can eventually be absorbed through the food chain and harm human health [7]. The continuous development of industrialization and agriculture of human society is accompanied by the emergence of soil heavy metal pollution. Activities such as industrial waste discharge, mining, agricultural use of sludge, and application of Zn-containing fertilizers lead to excessive accumulation of Zn in soil [8, 9]. The alarms of plant Zn toxicity and soil Zn contamination have drawn the attention of the scientific community to measures to mitigate excess Zn-stressed plants. Implementing of artificial auxiliary measures, such as plant hormone application and microbial symbiotic culture, can effectively improve plant tolerance without directly damaging the soil environment.

*Medicago sativa* L. (*M. sativa*) is a perennial leguminous with large biomass, high protein and fiber content, and is a high-quality forage grass [10]. Its well-developed roots and ability to fix nitrogen are very conducive to improving soil fertility [10]. Moreover, it is resistant to drought, cold, salt, especially heavy metals, and has strong adaptability. *M. sativa* is a pioneer plant to repair contaminated soil in mining area [11, 12]. *M. sativa* can grow in acidic copper mine tailings in arid lands and enrich cuprum (Cu), lead (Pb), cadmium (Cd), Zn, and other heavy metals [13]. *M. sativa* can also regulate osmotic pressure and redox reaction to activate heavy metal detoxification mechanism to cope with Pb poisoning [14, 15]. The application of sodium bicarbonate and citric acid can significantly improve the hyperaccumulation and purification efficiency of Cd, Cu, mercury (Hg), Pb, and Zn in *M. sativa *[16]. *M. sativa* has good tolerance and enrichment ability to heavy metals.

Phytohormone application is considered to be one of the methods to improve plant tolerance to heavy metals [17]. Through the crosstalk of phytohormone, osmotic pressure regulation, oxidative stress, heavy metal transport and other signal transduction pathways, the application of phytohormones can mediate the mechanism of heavy metal detoxification in plants [18]. Gibberellic acid (GA) plays an important role in plant growth and development, including regulating cell elongation and division, breaking dormancy and reproductive development [19]. And now the mechanism of GA alleviating abiotic stress in plants has been gradually revealed. GA could be counteracted the toxicity of Cd and Pb to *Vicia faba* L. plants [20]. GA can reduce the harmful effect of low concentrations of Pb and Cd to *Chlorella vulgaris *[21]. The inhibited levels of chromium (VI) on *Pisum sativum* (L.) seedlings growth, nitrogen assimilation and antioxidant system could be alleviated by GA [22]. Cd stress resulted in in lipid peroxidation and nitric oxide accumulation in *Arabidopsis thaliana* roots, and GA could effectively reduce them [23].

Brassinosteroids (BR) regulates various physiological functions such as embryogenesis and seed germination, cell division and elongation, differentiation and growth of pollen tubes, and leaf senescence and death [24]. Notably, BR also regulates plant resistance to heavy metal toxicity. BR pre-soaking treatment alleviated the peroxidative caused by Cd and chromium (Cr) in radish (*Raphanus sativus* L. var. Pusa Chetki) [25]. Exogenous application of BR promoted the development of tomato (*Lycopersicon esculentum*) seedlings under Cd stress by improving the activities of photosynthetic system and antioxidant system [26]. BR pre-germination treatment improved plant biomass production by blocking Cu uptake and accumulation [27]. At present, GA and BR have mature and common synthetic pathways. Therefore, it is possible to use GA and BR to assist plant remediation of Zn-contaminated soil easily and efficiently.

The effects and coping measures of Zn deficiency on plants have been extensively reported. However, there are few studies on BR and GA on Zn uptake and transport in plants under excessive Zn. With *M. sativa* as research material, we designed pot experiments, treated seedlings with different Zn concentrations, and applied exogenous GA and BR treatments of the same concentration to observe and measure the physiological and biochemical performance and genes expression patterns, to explore effects of BR and GA on Zn toxicity and transport in plants.

## Materials and Methods

### Plant materials, Zn treatment and phytohormone application

The *M. sativa* seeds, cultivar Giulia, were surface-disinfected with 3% sodium hypochlorite for 20 min, and then rinsed with sterile water. The seeds were spread on the sterilized sand, poured into sterile water to cover the surface of the sand, covered with plastic wrap, and placed in a 25°C incubator for dark cultivation. After 3 days, the seeds were germinated, and then transferred to the growth chamber. Growth chamber conditions were 16 h of light (7:00 – 23:00, 25°C, Relative Humidity 60%) and 8 h of darkness (23:00 – 07:00, Relative Humidity 60%, 22°C). When the seedlings had 4 cotyledons, they were transplanted into square pots (7 cm × 7 cm × 7 cm) filled with sterilized sand and began to be irrigated with the completed Hoagland nutrient solution [28]. The control group was treated with nutrient solution with no modification. Zn treatment was changed from 0.8 mM ZnSO47H2O in Hoagland's nutrient solution to 1 mM and 2 mM. The 24-Epibrassinolide (24-epiBL) and GA powder was dissolved in a small amount of anhydrous ethanol, then diluted to 20 mM in sterile water and stored at -20℃. 20 mM 24-epiBL and GA were diluted with nutrient solution to 100 nM respectively to treatment. Zn stress (1 mM or 2 mM) × Phytohormone (BR or GA) two-factor randomized block design was used, with 9 plant biological replicates per treatment.

After 45 days of treatment, *M. sativa* plants were harvested. And their aboveground and underground biomass were weighed, and then the samples were frozen in liquid nitrogen and transferred to a—80℃ refrigerator for storage.

### Biochemical index determination

The samples were dried in an oven at 80°C to constant weight, then weighed (0.1 g or less) and ground to fine powders. Then the samples were completely digested with 5 mL ternary mixtures of HNO3: H2SO4: HClO4 (10:1:4 (V/V/V)). Appropriate amounts of Zn element standard stock solution were diluted step by step to draw a standard curve. The absorption peaks were determined using the inductively coupled plasma atomic emission spectrometer (ICP, 710-ES, Varian, USA). Finally, the Zn concentration of the samples solution was calculated according to the standard curve, and the Zn mass fraction of the samples was calculated based on the mass.

We determined the content of catalase (CAT), peroxidase (POD), superoxide dismutase (SOD), superoxide anion (OFR) and malondialdehyde (MDA) in samples by performing with CAT Kit, POD Kit, SOD Kit, OFR Kit and MDA Kit (Solarbio, Beijing, China) according to the manufacturer's protocol.

### Genes expression

Total RNA was isolated by the Plant RNA Kit (Omega Bio-Tek, Georgia, America). cDNA was synthesized from 1 µg of RNA with ChamQ Universal SYBR qPCR Master Mix (Vazyme, Nanjing, China), and Real-time PCRs were performed using HiScript III RT SuperMix for qPCR (+ gDNA wiper) (Vazyme, Nanjing, China) according to the manufacturer's instructions. The primers design refers to the results of Cardini et al. 's study in 2021 [29], and the detailed primers sequences were presented in table S1.

### Statistical analysis

All data were analyzed using SPSS 16 software (SPSS, Chicago, USA) for two-factor analysis of variance (ANOVA) (including Brown-Forsythe test and Bartlett test) and Tukey’s multiple comparisons test. The data were presented as t mean ± standard deviation (SD) of different replicates. And different letters indicate a significant difference at *p* < 0.05.

## Results

### BR and GA application improved M. sativa growth under Zn stress

**Fig. 1:**
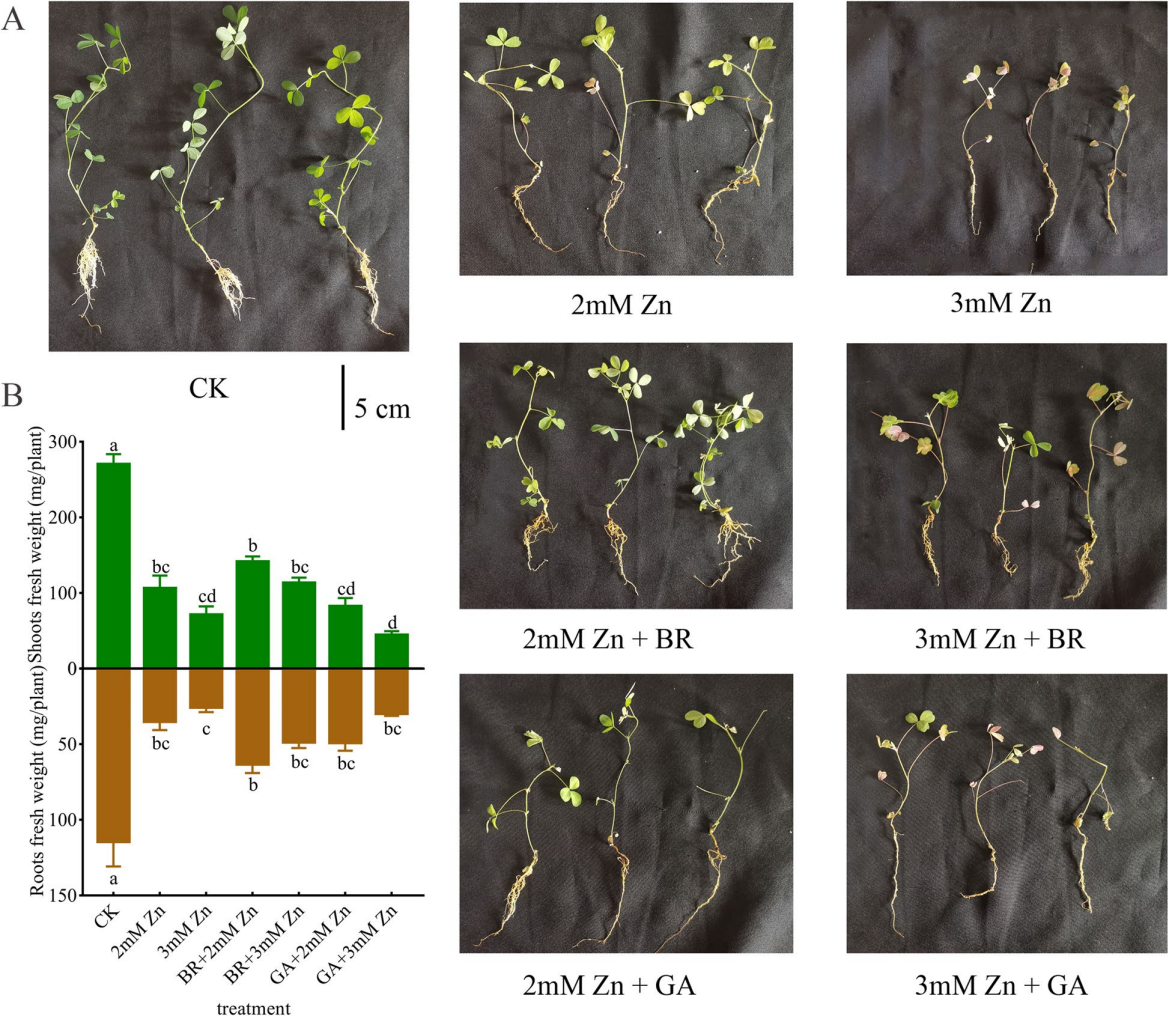
Effects of Zn stress, BR and GA application on growth of *M. sativa.*

The effect of Zn, BR and GA treatments on *M. sativa* growth was assessed (Fig. 1). The results showed that Zn treatment (1 mM and 2 mM) had significantly adverse effects on the biomass and phenotype of *M. sativa* compared to the control group. The biomass under Zn stress, both in roots and shoots, was significantly lower than in untreated plants (Fig. 1A). Moreover, under Zn stress, *M. sativa* plants type was weaker and smaller, with the old leaves yellowing and wilting, fewer branches, and shorter roots (Fig. 1B).

*M. sativa* seedlings were treated with Zn, BR and GA for 7weeks. (A) the phenotype of seedlings under different treatments before sampling. Scale bars, 5 cm. (B) The root and shoots biomass of *M. sativa* upon different treatments as described above. The fresh weight of roots or shoots is expressed as mg/plant. Analysis of variance (ANOVA) (including Brown-Forsythe test and Bartlett test) and Tukey’s multiple comparisons test were performed on the data of 6 biological replicates (*n*= 6). Vertical bars represent mean ± standard deviation (SD). Averages with the different letter are significantly different at *p* < 0.05.

Under the treatment of 100 nM BR or GA, the roots and shoots biomass of *M. sativa* were higher than those of plants under the same Zn stress. In particular, under the same concentration of Zn stress, the shoots biomass treated with 100 nM BR was significantly higher than that treated with 100 nM GA. Consistently, BR and GA showed the same trend for the improvement of *M. sativa* roots biomass, even though the difference between BR and GA application did not reach the level of significance (Fig. 1B). The application of BR and GA made *M. sativa* leaves return to green to some extent, and the plant type also partially recovered (Fig. 1A). These results indicated that the application of BR or GA could improve the growth of *M. sativa* under Zn stress. And under the same Zn stress conditions, the same concentration of BR may have better mitigation effect on plants than GA.

### BR and GA application affected Zn uptake and transport in M. sativa shoots and roots

**Fig. 2:**
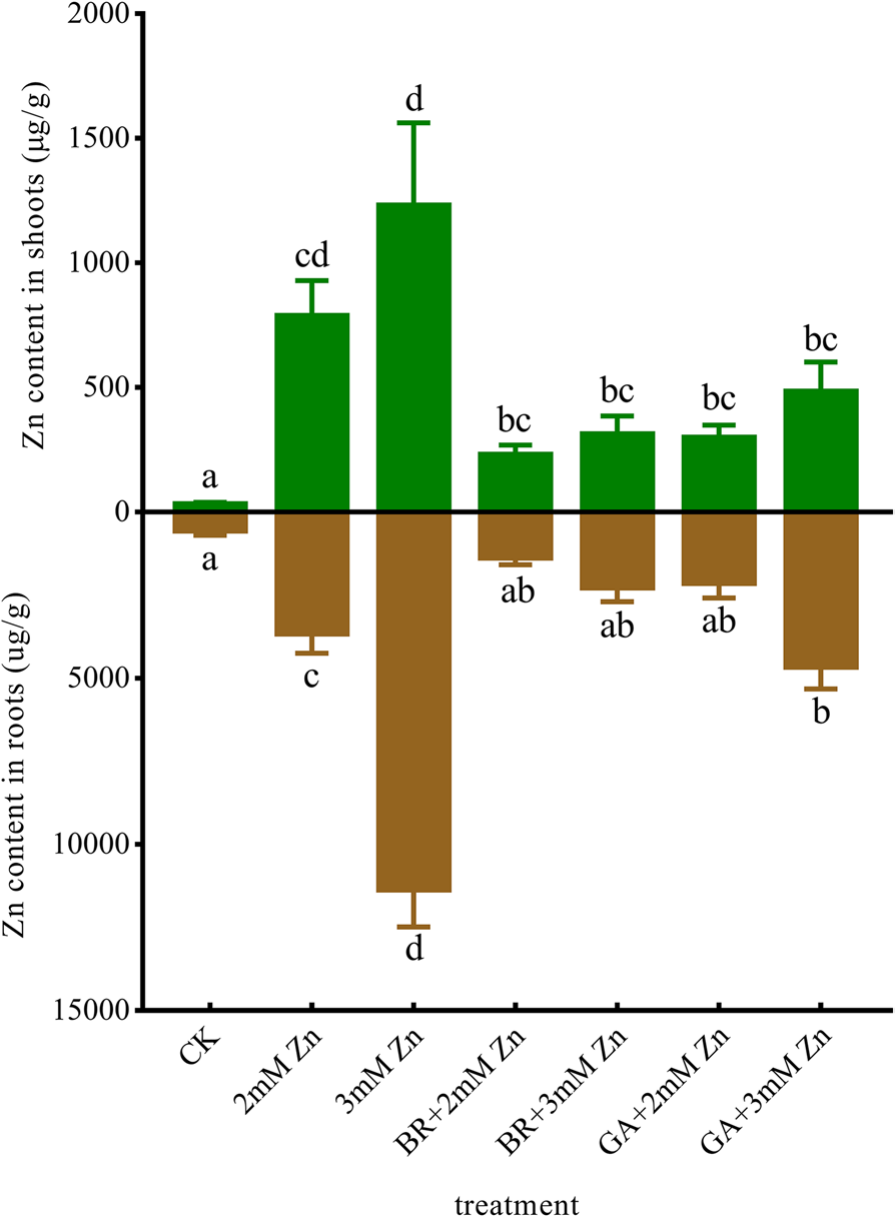
Effects of Zn, BR and GA application on Zn enrichment and transport in *M. sativa. *ANOVA (including Brown-Forsythe test and Bartlett test) and Tukey’s multiple comparisons test were performed on the data of 3 biological replicates (*n *= 3). Vertical bars represent mean ± SD. Averages with the different letter are significantly different at *p* < 0.05

Zn content was measured in the *M. sativa* roots and shoots after Zn, BR and GA treatments (Fig. 2). In control plants, Zn levels between roots and shoots were similar. The Zn content in *M. sativa* roots under 2 mM Zn treatment was about 5 times more than that in shoots. And under other treatments, it has reached over 7 times in shoots (Fig. 2). Compared with control plants, Zn content in *M. sativa* was significantly increased under 2 and 3 mM Zn stress. And the roots Zn content under 3 mM Zn was significantly higher than that under 2 mM Zn. The same trends were also shown in the shoots, but the differences were not significant (Fig. 2). These results suggested that *M. sativa* mainly enriched Zn in roots to alleviate Zn toxicity in the aboveground tissues to tolerate stress and maintain normal growth.

BR and GA application significantly reduced Zn content in roots under all the Zn treatments. The application of BR and GA also reduced the Zn content in shoots under all the Zn stress, though the difference was not significant under 2 mM Zn stress. Under the same Zn concentration treatment, Zn content in roots and shoots of *M. sativa* treated with BR was lower than with GA (Fig. 2). These indicated that the application of BR and GA significantly alleviated Zn stress on *M. sativa*, possibly by reducing roots enrichment. In addition, with the same concentration of BR and GA, the effect of BR on inhibiting excessive Zn accumulation in roots was stronger.

### BR and GA application attenuated oxidative stress caused by excess Zn in M. sativa

**Fig. 3:**
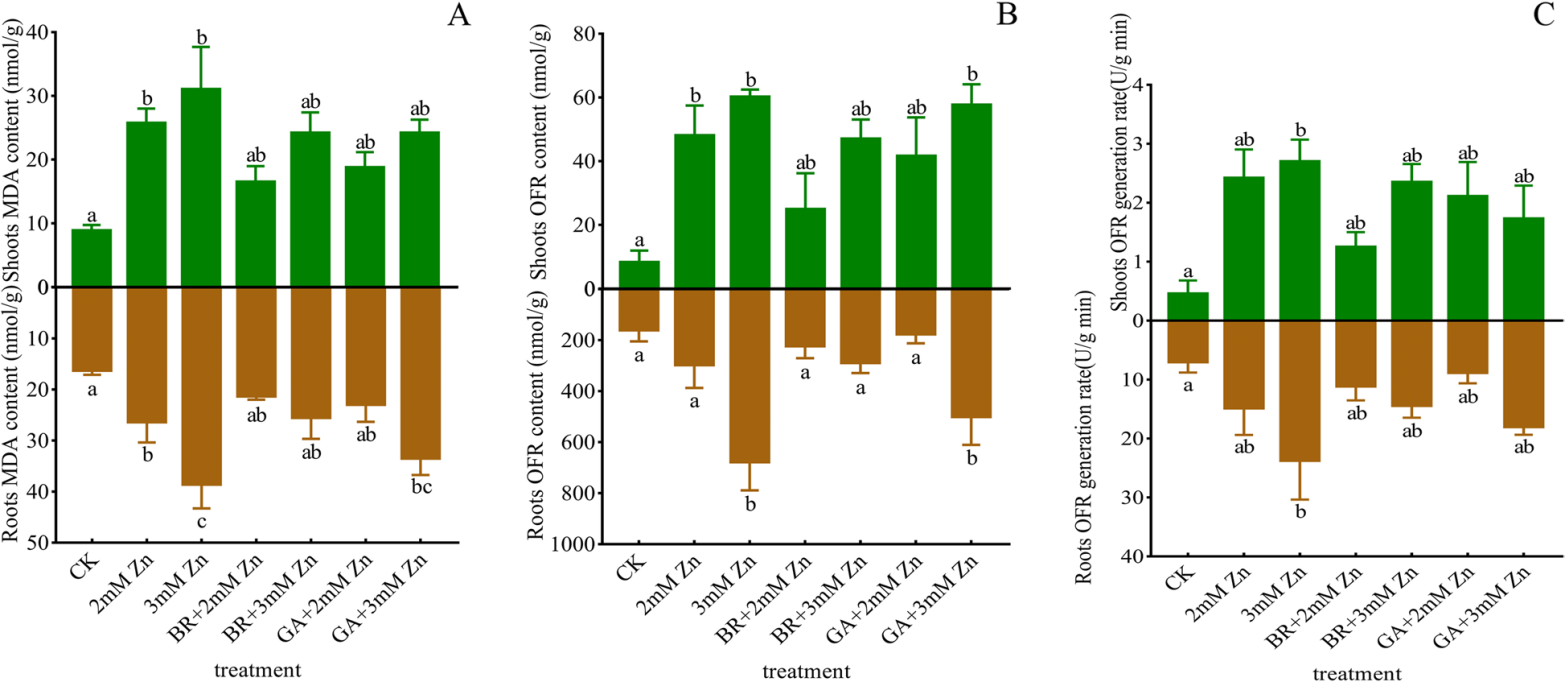
Effects of Zn, BR and GA application on peroxide levels in *M. sativa. *Analysis of MDA content (A), OFR content (B) and its production rate (C) activity in *M. sativa* roots and shoots after 7 weeks of treatment. ANOVA (including Brown-Forsythe test and Bartlett test) and Tukey’s multiple comparisons test were performed on the data of 3 biological replicates (*n* = 3). Vertical bars represent mean ± SD. Averages with the different letter are significantly different at *p* < 0.05

Lipid peroxides and antioxidant enzymes were regulated by Zn, BR, and GA treatments. Compared with control *M. sativa* plants, MDA content in roots and shoots increased significantly under 2 mM and 3 mM Zn stress. BR and GA reduced the MDA level of plants under Zn stress, and BR-treated plants had lower MDA content than GA-treated plants, even though these differences were not significant. Moreover, MDA content in roots was higher than shoots under all treatments (Fig. 3A).

The OFR activity and production rate of *M. sativa* plants under Zn stress were higher than those of control group. And the OFR activity and production rate of roots treated with 3 mM Zn were significantly different from that of control plants. BR and GA reduced the OFR activity and production rate of Zn-stressed plants to levels that were not significantly different from the control group. A similar pattern of OFR and its production rate was observed in shoots. In particular, the OFR activity difference of the shoots between 2 mM Zn stress and the control group was also significant. In addition, the ORF activity and production rate of plants treated with BR were lower than those treated with GA (Fig. 3 BC).

**Fig. 4:**
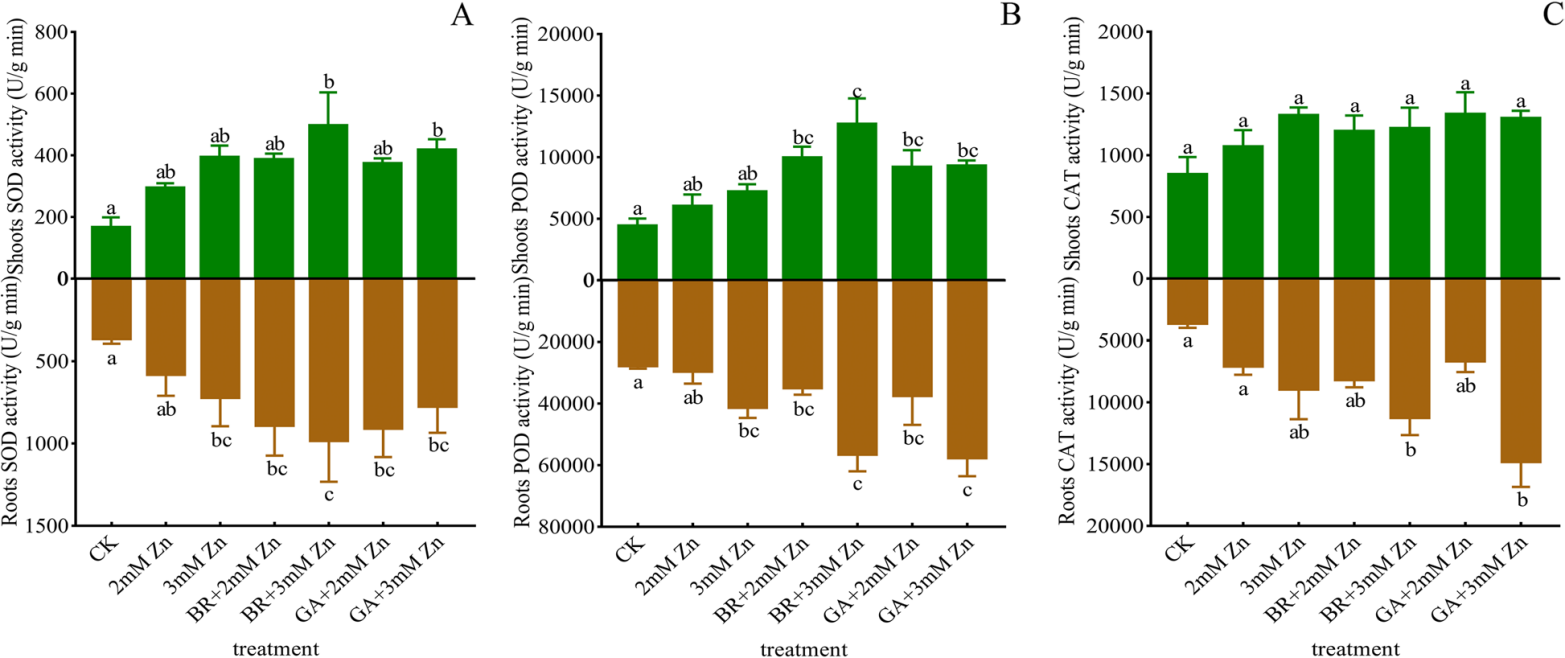
Effects of Zn stress, BR and GA application on antioxidant enzyme activity of *M. sativa. *Analysis of SOD (A), POD (B), and CAT (C) activities in *M. sativa* roots and shoots after 7 weeks of treatment. ANOVA (including Brown-Forsythe test and Bartlett test) and Tukey’s multiple comparisons test were performed on the data of 3 biological replicates (*n* = 3). Vertical bars represent mean ± SD. Averages with the different letter are significantly different at *p* < 0.05

The effects of Zn stress, BR and GA on *M. sativa* roots or shoots were similar. 2 mM and 3 mM Zn increased SOD activity of *M. sativa*, but the difference was not significant compared with the control group. BR and GA increased SOD activity of *M. sativa* plants under Zn stress. And the SOD activity of *M. sativa* plants under 3 mM Zn + BR was higher than that of 3 mM Zn + GA (Fig. 4A). The effects of different treatments on POD activity were like that of SOD activity. In particular, under the same Zn concentration, no matter roots or shoots, BR-treated plants showed higher POD activity than GA (Fig. 4B).

There was no significant difference in CAT activity of *M. sativa* shoots under different treatments. But, Zn-treated plants shoots CAT activity was higher compared to control. Contrary to the performance of shoot CAT activity, the CAT activity of roots under different treatments showed obvious differences. Under the same Zn concentration, the application of BR and GA increased the CAT activity of roots. It is worth mentioning that the CAT activity of roots was much higher than that of shoots (Fig. 4C).

### BR and GA regulated the transcription level of Zn transporters

Zn, BR and GA mediated the expression of *M. sativa ZRT-IRT-like protein* (*MsZIP*) related to Zn in and out of cells.

**Fig. 5:**
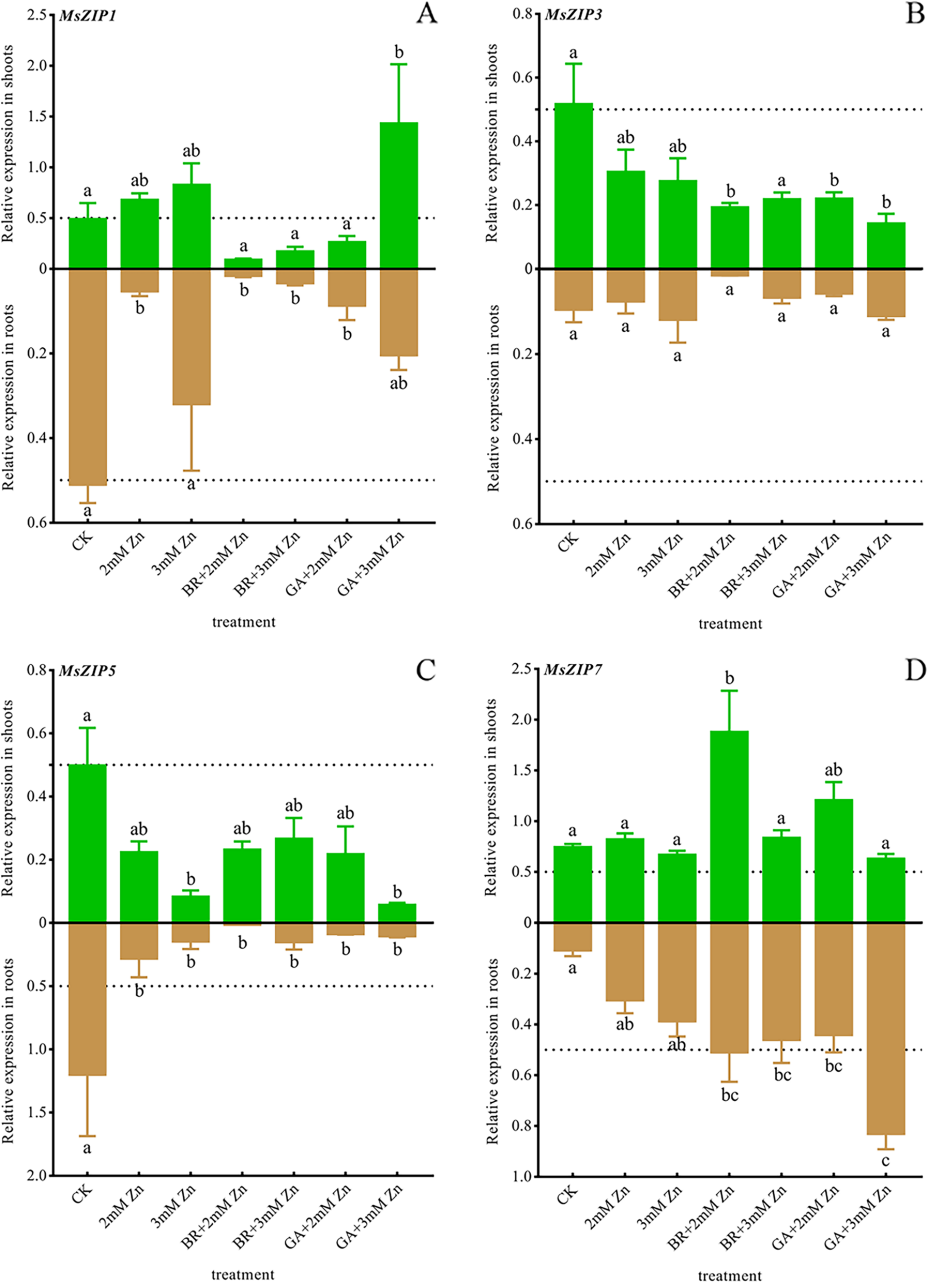
Effects of Zn stress, BR and GA on the expressions of Zn-flow related genes.

Zn treatments down-regulated *MsZIP1*, *MsZIP3*, and *MsZIP5* expressions. And this downregulation was reinforced by BR and GA applications. In the control group, the expressions of *MsZIP1* were similar in roots and shoots. However, Zn stress significantly down-regulated the expressions of *MsZIP1* in roots. Under the same Zn concentration, BR significantly reduced the expressions of *MsZIP1* in roots, while GA insignificantly down-regulated its expressions. There was no significant difference in expressions of *MsZIP1* in *M. sativa* shoots under different treatments. And BR and GA down-regulated the expression of *MsZIP1* in shoots under 2 mM Zn (Fig. 5A). Different from *MsZIP1*, the expressions of *MsZIP3* were higher in shoots than in roots (Fig. 5B). And, the expressions of *MsZIP3* were higher in roots than that in shoots. Compared with the control group, Zn stress, BR and GA did not significantly down-regulate the expressions of *MsZIP5* in roots and shoots (Fig. 5C). In contrast, Zn stress upregulated the expressions of *MsZIP7*, though insignificantly. While this upregulation was reinforced by BR and GA applications. In addition, *MsZIP7* expressions in shoots were higher than that in roots (Fig. 5D).

Total RNA was isolated from *M. sativa* roots and shoots. The *MsEF1α* from *M. sativa* was used as the housekeeping gene for normalization. After 7 weeks of treatment, the expressions of *MsZIP1/3/5/7* (A-D), that involved in Zn flow in and out of cells, was determined in the roots and shoots of *M. sativa*. ANOVA (including Brown-Forsythe test and Bartlett test) and Tukey’s multiple comparisons test were performed on the data of 3 biological replicates (*n *= 3). Vertical bars represent mean ± SD. Averages with the different letter are significantly different at* p* < 0.05.

Zn, BR and GA mediated the expression of *M. sativa metal tolerance protein 1* (*MsMTP1*) and *zinc-induced facilitators 1* (*MsZIF1*) associated with Zn accumulation in vacuoles.

**Fig. 6:**
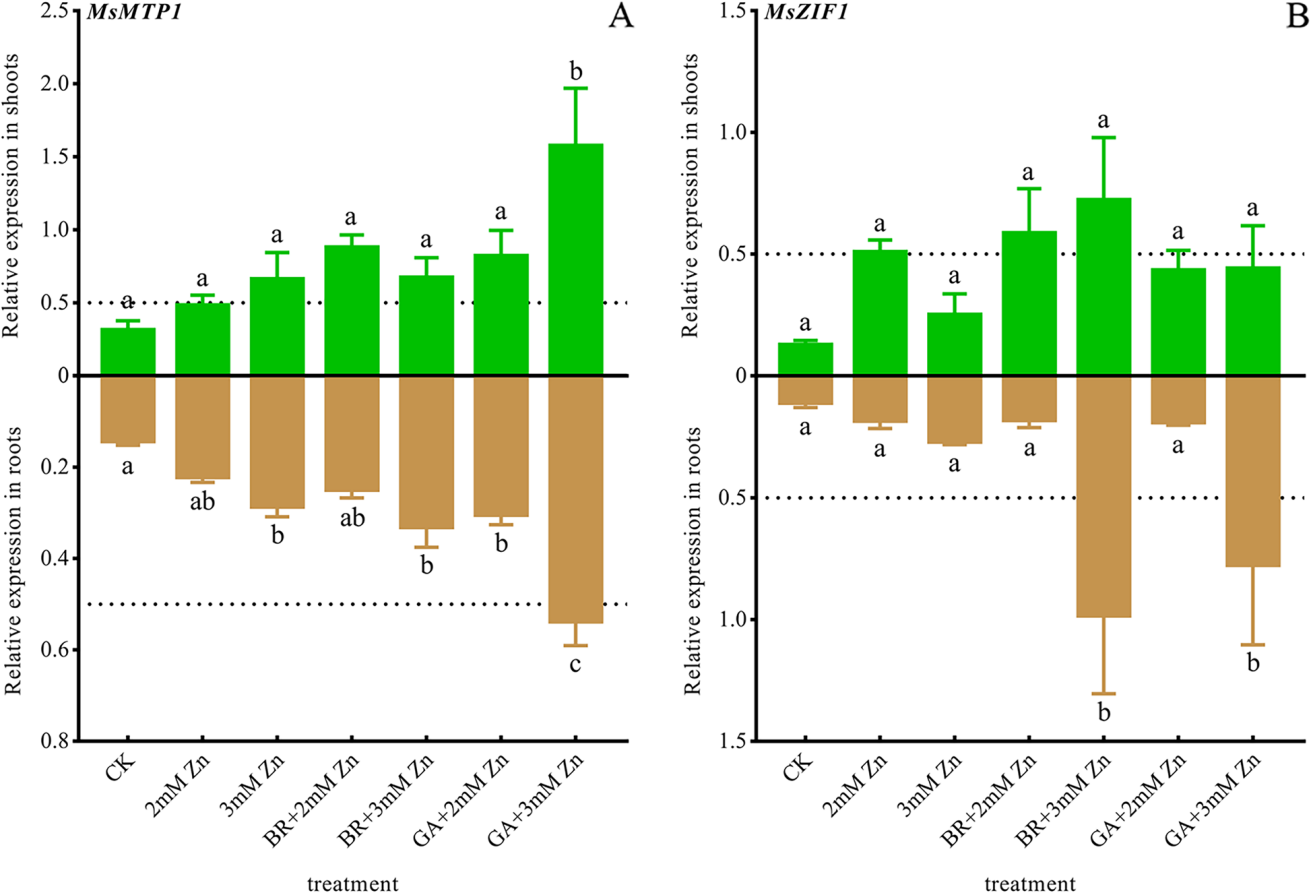
Effects of Zn stress, BR and GA on the expressions of genes related to Zn-efflux. Total RNA was isolated from *M. sativa* roots and shoots. The *MsEF1α* gene from *M. sativa* was used as the housekeeping gene for normalization. The expressions of *MsMTP1* (A) and *MsZIF1* (B) genes involved in Zn efflux and vacuolar storage were determined in roots and shoots of *M. sativa* after 7 weeks of treatment. ANOVA (including Brown-Forsythe test and Bartlett test) and Tukey’s multiple comparisons test were performed on the data of 3 biological replicates (*n* = 3). Vertical bars represent mean ± SD. Averages with the different letter are significantly different at *p* < 0.05

*MsMTP1* was more strongly expressed in shoots than in roots, but there was no significant difference between different treatments in shoots. Zn stress up-regulated the expressions of *MsMTP1* on shoots and roots. Further, BR and GA promote this up-regulation. The expressions of *MsMTP1* in *M. sativa* shoots under 3 mM Zn and GA application were significantly higher than that in the control plants. And the expressions of *MsMTP1* in roots under 3 mM Zn and its application with BR or GA were significantly higher than that in the control plants (Fig. 6A).

Unlike *MsMTP1*, the expressions of *MsZIF1* were similar in roots and shoots. Zn stress, BR and GA also up-regulated the expressions of *MsZIF1* in shoots, but there was no significant difference among different treatments. The expressions of *MsZIF1* in roots under 3 mM Zn and BR or GA was significantly higher than that under other treatments (Fig. 6B).

Zn, BR and GA mediated the expression of *M. sativa heavy metal transporters 4, yellow stripe-like protein 1, and nicotianamine synthase 1* (*MsHMA4*, *MsYSL1* and *MsNAS1*) related to Zn chelation and long-distance transport.

**Fig. 7:**
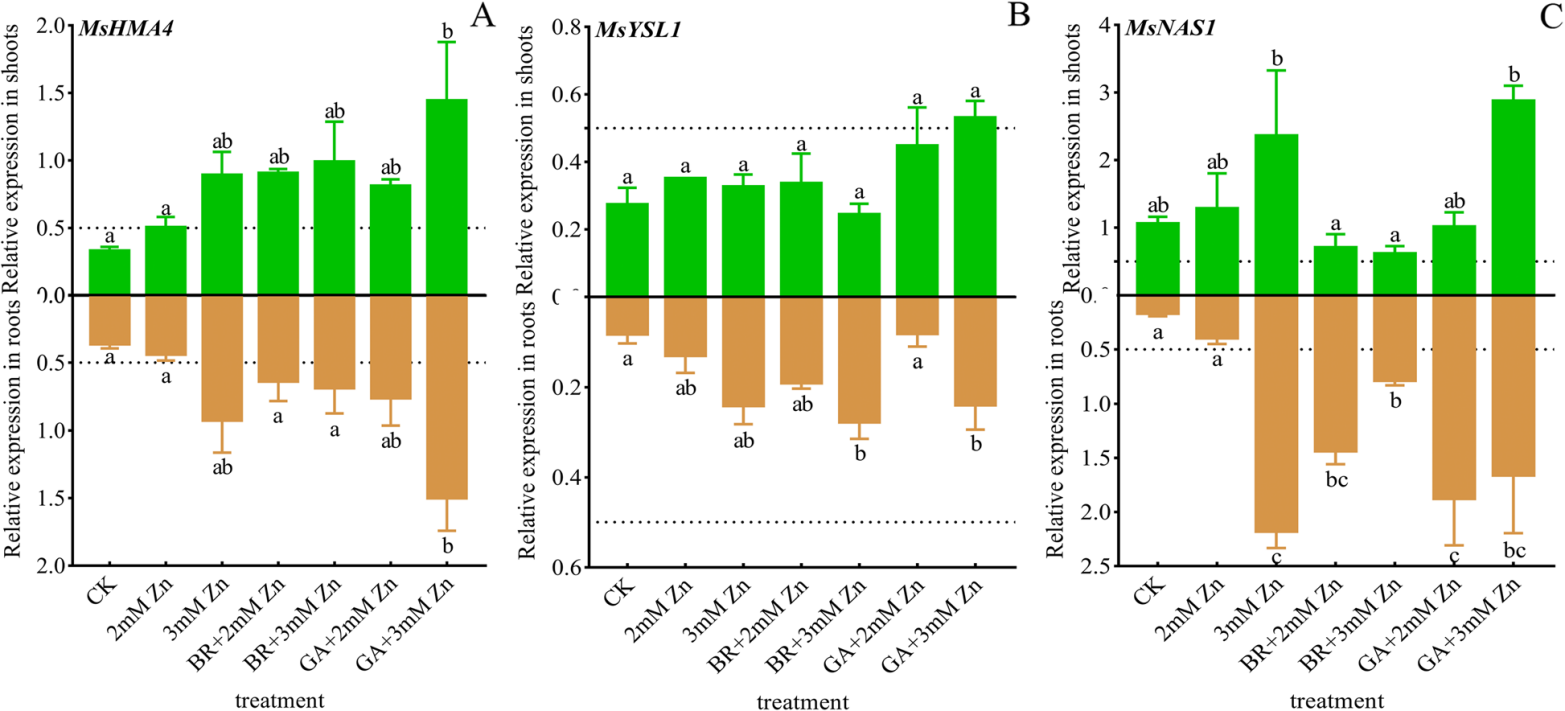
Effects of Zn, BR and GA on the expressions of Zn chelation and transport genes. Total RNA was isolated from M. sativa roots and shoots. The *MsEF1α* gene from *M. sativa* was used as the housekeeping gene for normalization. The expressions of *MsHMA4* (A), *MsYSL1* (B) and *MsNAS1* (C) genes involved in Zn chelation and long-distance transport were determined in the roots and shoots of *M. sativa* after 7 weeks of treatment. ANOVA (including Brown-Forsythe test and Bartlett test) and Tukey’s multiple comparisons test were performed on the data of 3 biological replicates (*n* = 3). Vertical bars represent mean ± SD. Averages with the different letter are significantly different at *p* < 0.05

*MsHMA4* was strongly induced to express in both roots and shoots. Zn stress, BR and GA up-regulated the expressions of *MsHMA4*. BR and GA up-regulated *MsHMA4* expressions in shoots under the same Zn concentration. And under 3 mM Zn + GA, *MsHMA4* expressions were significantly higher than that under control. In particular, the expressions of *MsHMA4* in roots showed consistent patterns (Fig.7A).

On the whole, the expressions of *MsYSL1* in shoots were slightly higher than that in roots. Zn stress, BR and GA up-regulated the expressions of *MsYSL1*. The expression level of *MsYSL1* in roots under 3 mM Zn and BR or GA treatment was significantly higher than that in the control. Besides this, the differences among other treatments were not significant (Fig.7B).

*MsNAS1* was strongly expressed in both roots and shoots. And the expressions of *MsNAS1* in root was higher than that in shoots in control group. Zn stress, BR and GA up-regulated the expressions of *MsNAS1*, significantly. The expressions of *MsNAS1* in roots under 2 mM Zn was significantly upregulated by BA and GA. The expressions of *MsNAS1* in shoots was up-regulated under Zn stress (Fig. 7C).

## Discussion

### BR and GA alleviated oxidative stress and growth inhibition of *M. sativa* under Zn stress

Zn accumulates in the soil and is absorbed and used by plants through their roots [30]. After Zn ions are absorbed by roots, some of them are chelated and stored in vacuoles, some are transported or diffused between cells, and some are transported to xylem step by step [31, 32]. And then, Zn ions are chelated in the xylem by nicotinamide, transported over long distances as free cations, and finally unloaded in mesophyll cells [33, 34]. Then, some of them are chelated and stored in vacuoles or transported and diffused between cells, and some of them are transported to the reproductive organs through the loading and unloading of phloem [35]. With excess Zn, the above series of plant Zn uptake and transport networks are responded to and regulated.

We found that excess Zn significantly inhibited the accumulation of *M. sativa* biomass. And Zn stress significantly increased MDA and OFR content and production rate. Meanwhile, the activities of CAT, SOD and POD were increased under Zn stress. These also confirmed that oxidative stress caused by excessive heavy metals can significantly inhibit plant growth and biomass accumulation [36, 37]. The results of correlation analysis also showed that Zn accumulation in plants had a significant negative correlation with biomass, and a significant positive correlation with MDA, OFR, CAT, SOD, and POD activities (Table S2-4). Besides, these indicators are also correlated with each other (Table S2-4). We speculate that this is a chain reaction of growth inhibition and oxidative stress triggered by increased Zn accumulation in plants. It is worth mentioning that BR and GA significantly eased this inhibition. Under the same Zn stress, BR and GA increased *M. sativa* biomass. Moreover, the application of BR and GA further decreased the content of lipid oxides (MDA and OFR), and further increased the activities of antioxidant enzymes (CAT, SOD, POD).

BR significantly increased plant heights, chlorophyll content and enzyme activity of *Chlorella vulgaris* under Cd stress [38]. Foliar spraying of BR significantly enhanced the biomass, antioxidant enzyme activities and proline content of mung bean (*Vigna radiata* L. Wilczek) under aluminium (Al) stress [39]. Exogenous BR application up-regulated the transcription levels of antioxidant enzyme-related genes in rice in rice (*Oryza sativa* L.), Maize (*Zea mays* L.), and Pepper (*Capsicum annuum*) under heavy metal stress [40–42]. GA treatment up-regulated the activity of various oxidoreductases to alleviate chromium (VI) phytotoxicity in *Pisum sativum* (L.) [22]. And GA treatment at an appropriate concentration increased proline and antioxidant enzyme levels of spinach (*Spinacia oleracea* L.) to alleviate Cu stress [43]. These evidences indicated that BR and GA play an important role in alleviating heavy metal toxicity and reducing oxidative stress response to promote plant growth. It is worth mentioning that our study showed that BR application at the same concentration was better than GA in this regard under the same Zn concentration stress.

### BR and GA reduced the excessive accumulation of Zn in *M. sativa*

The results showed that over five times as much Zn content was accumulated in the roots as in the shoots. The roots biomass was reduced more than the shoots because of the more severe stress caused by direct exposure to excessive Zn [36, 44]. Similarly, Namdjoyan et al. found that the roots of safflower (*Carthamus tinctorius* L.) seedlings accumulated more Zn than those in shoots [37]. These support the view that plants accumulate much more heavy metals in the roots to reduce the toxicity to the aboveground and thus enhance their tolerance.

It has been reported that BR and GA reduce the accumulation of other heavy metals (such as Pb, Cu, Cr, Cd, and Zn) in plants [18, 45]. GA can improve plant tolerance to heavy metal toxicity via reducing accumulation or accelerating excretion [46, 47]. BR and GA application significantly reduced excessive Zn accumulation in *M. sativa* roots and shoots. And the application of BR and GA decreased the lipid peroxides of *M. sativa* roots to a greater extent than shoots, and improved the activity of antioxidant enzymes more strongly in roots than in shoots. Particularly, the same concentration BR inhibited the excessive accumulation of Zn more strongly than GA.

### BR and GA regulated heavy metal transporters expression patterns in *M. sativa* with excess Zn

The Neighbor-joining (NJ) phylogenetic trees of *ZIP*, *MTP*, *ZIF*, *HMA*, *YSL* and *NAS* gene sequences of *M. sativa* and other plant species have been inferred, including *Medicago truncatula*, *Arabidopsis thaliana* (L.) Heynh., *Glycine max* (L.) Merr., *Hordeum vulgare* L., *Oryza sativa* L., *Triticum aestivum* L., and *Zea mays* L. [29]. First, *MsZIP1-7* gene sequences and *MtZIP1-7* existed as lineal homologues. In addition, the sequences of *MsZIP1/3/5* were closely related to those of *AtZIP3/4/7*, *OsZIP4/5/8*, *ZmZIP4/5/7*, and *HvZIP3/5/7*. Particularly, the sequence of *MsZIP1*, *OsZIP1/2*, *ZmZIP2* and *HvZIP1/2* were similar to each other. As regards *MTP*, the sequence of *MsMTP1* was also related to those of *GmMTP1*, *AtMTP1/2/3*, *ZmMTP1*, *HvMTP1* and OsMTP1/2/3. As regards *ZIF*, the sequence of *MsZIF1* clustered with the sequences of *AtZIF1/2*, *ZmZIF1* and *GmZIF1*. As regards *HMA*, the sequence of *MsHMA1* was also related to *AtHMA1/2/3*. Similarly, the sequence of *MsYSL1* was most similar to those of *AtYSL1/2/3*, *ZmYSL1* and *OsYSL1* and the sequence of *MsNAS1* was most similar to those of *AtNAS1/2/3*, *ZmNAS1* and *OsNAS1* [29].

ZIP family genes located on the plasma membrane are involved in the flow of Zn in and out of cells [48]. And they can transport a variety of metal ions (Fe, Zn, Cd, Cu, etc.) and are subject to complex metal-dependent post-translational regulation [49]. *AtZIP3/4/7*, *OsZIP4* and *HvZIP3/5/7* have been identified as Zn transporters on the plasma membrane, which are strongly induced by Zn deficiency and mediate the flow of Zn into root cells [50–53]. It is emphasized that *ZmZIP4/5/7 wa*s strongly induced by Zn deficiency, and was highly expressed mainly in shoots, and was significantly inhibited under Zn stress [54]. These reports suggested that the homologous sequence closely related to MszZIP1/3/5 has the function of Zn uptake into root cells and is actively expressed by Zn deficiency and possibly inhibited by Zn excess. Consistently, Zn stress down-regulated the expressions of *MsZIP1/3/5* in *M. sativa*. And BR and GA enhanced the down-regulations. Conversely, *ZmZIP2* and *HvZIP2* are not induced by Zn deficiency, and *ZmZIP2* is substantially induced by Zn excess. This is consistent with *MsZIP7*. They may be involved in the outflow of Zn from the cell. These results suggested that, to resist Zn stress, plants reduce intracellular Zn accumulation by inhibiting the expressions of genes regulating Zn influx and promoting the expressions of genes regulating Zn outflow. And BR and GA actively regulate this process. In addition, the down-regulation of *MsZIP1/5* expressions induced by Zn stress was more extensive in roots than in shoots. This may be because plants already limit Zn uptake and transfer in the roots, so Zn stress in shoots is less intense than in roots.

Cellular vacuoles can store excess Zn in response to Zn poisoning or as a source of Zn deficiency. ZIF1 and MTP1 are involved in the transport of Zn—organic acids complexes into vacuoles. *AtMTP1*/*3* and *AtZIF1/2* actively regulate Zn chelation and vacuolar storage to enhance Zn tolerance in *Arabidopsis thaliana* [55–57]. Noticeably, *AtMTP1* was strongly expressed in both young leaves and seedling roots, while *AtMTP3* was strongly expressed only in roots when Fe deficiency and Zn excess occurred [55]. Consistently, Zn stress upregulated the expressions of *MsMTP1* and *MsZIF1*, and BR and GA promoted the upregulation. There are few reports on the functional characterization of MTP1 and ZIF1 homologous sequences in Zn excess. However, the up-regulation of *MsMTP1* and *MsZIF1* enhances the tolerance of *M. sativa* to Zn toxicity by enhancing chelating ability to excess Zn. In addition, up-regulation of *MsMTP1* and *MsZIF1* in roots may reduce Zn transport to shoots. Notably, BA and GA enhanced the up-regulation of *MsMTP1* expression. In summary, BR and GA levels promote Zn detoxification pathways.

NAS regulates niacinamide and heavy metal chelation [58], and HMA and YSL regulate ectopic heavy metal in xylem, phloem or cell to cell [32, 59]. *AtHMA2/3/4* can transfer Zn from root cells to xylem cells and are associated with ectopic Zn transfer from roots to shoots. Overexpression of *AtHMA3* enhanced the tolerance of Cd and Zn in *Arabidopsis thaliana*, and increased the accumulation of Zn in shoots and roots [32]. Similarly, the expressions of *MsHMA3* in *M. sativa* shoots and roots was up-regulated when exposed to Zn stressing. This upregulation promotes Zn transport to xylem to alleviate Zn toxicity. Notably, the upregulation was amplified by the application of BR and GA, further mitigating the Zn toxic.

It has been widely reported that NAS and YSL are regulated by Fe levels. Fe deficiency induced up-regulation of *ZmNAS1* and *ZmNAS2* expressions in maize roots [ 32]. However, *ZmNAS3*, *OsNAS1/2*, *AtYSL1/2/3*, *OsYSL2* and *ZmYSL1* were induced by excessive Fe [ 60]. In view of this, the alleviating effect of Fe abundance on Zn toxicity has also been reported. *AtNAS2*, *ATYSL1/3* and *OSYSL2/16/18* regulated the long-distance transport of Zn in xylem and phloem, and affect the accumulation of Zn in roots [ 53]. Excessive Zn induced up-regulation of *MsYSL1* and *MsNAS1*. These results indicated that the Zn chelating capacity of *M. sativa* was enhanced and xylem transfer was increased. These processes are beneficial for plants to alleviate excessive Zn toxicity. In addition, chelating substrates (plant ferrite group or niacinamide) also affect the chelation and transport of heavy metals. There was little difference in the expression of *MsYSL1* in shoots under different treatments. Whether it was affected by the level of Zn chelating substrate in cells requires more research experiments.

## Conclusion

Phytohormone play an important role in plant resistance to stress. Our results suggested that, BR and GA can restore the growth damage caused by zinc poisoning. And BR and GA can also promote plant antioxidant system response and heavy metal detoxification mechanism. Interestingly, the same concentration of BR alleviates plant heavy metal tolerance slightly better than GA. It is well known that plants will adjust the nutrient uptake or heavy metal enrichment efficiency of different tissues such as roots, leaves, stems, buds, flowers and fruits, to cope with heavy metal stress. These processes are involved in phytohormone. Therefore, a more detailed understanding of the detoxification mechanism of heavy metals in plants will be improved by combining the growth status of different tissues and nutrient uptake status. These processes are involved in phytohormone. Therefore, phytohormone changes combined with different tissue growth status and nutrient uptake status will improve the understanding of the detoxification mechanism of heavy metals in plants.

## Fundings

This work was supported by the Science and Technology Planning Project of Guangdong Province [Grant number: 201904020022], the Laboratory of Lingnan Modern Agriculture Project [Grant number: NZ2021025], and the National Natural Science Foundation of China [Grant number: 32071639].

## Supplementary Information

**Additional file 1:** **Table S1.** The primer sequences of the genes. **Table S2.** Spearman's Rho among different indexes such as zinc content, biomass and antioxidant enzymes. **Table S3.** Total variance explained of principal component analysis for all indicators. **Table S4.** Component Matrixa of principal component analysis for all indicators.

**Additional file 2:Table 1.** Effects of different treatments on *Medicago sativa* L. biomass (Fresh weight, mg/plant). **Table 2.** Effects of different treatments on *Medicago sativa* L. Zinc content (μg/g). **Table 3.** Effects of different treatments on *Medicago sativa* L. MDA content (nmol/g). **Table 4.** Effects of different treatments on *Medicago sativa* L. OFR content (nmol/g). **Table 5.** Effects of different treatments on *Medicago sativa* L. OFR generation rate (U/g min). **Table 6.** Effects of different treatments on *Medicago sativa* L. SOD acitivity (U/g min). **Table 7.** Effects of different treatments on *Medicago sativa* L. POD acitivity (U/g min). **Table 8.** Effects of different treatments on *Medicago sativa* L. CAT acitivity (U/g min). **Table 9.** Effects of different treatments on genes relative expression in *Medicago sativa* L.

## Data Availability

The raw data for this article can be accessed in the Supplementary materials.

## Abbreviations

Zn: Zinc
*M. sativa*: *Medicago sativa* L
Cu: Cuprum
Pb: Lead
Cd: Cadmium
Hg: Mercury
GA: Gibberellic acid
VI: Chromium
BR: Brassinosteroids
24-epiBL: 24-Epibrassinolide
Cr: Chromium
CAT: Catalase
POD: Peroxidase
SOD: Superoxide dismutase
OFR: Superoxide anion
MDA: Malondialdehyde
*ZIP1*: *ZRT-IRT-like protein 1*
*MTP1*: *Metal tolerance protein 1*
*HMA4*: *Heavy metal transporters 4*
*YSL1*: *Yellow stripe-like protein 1*
*NAS1*: *Nicotianamine synthase*
NJ: Neighbor-joining
